# The public health significance of prior homelessness: findings on multimorbidity and mental health from a nationally representative survey

**DOI:** 10.1017/S2045796024000659

**Published:** 2024-11-08

**Authors:** N. Chilman, P. Schofield, S. McManus, A. Ronaldson, A. Stagg, J. Das-Munshi

**Affiliations:** 1 Department of Psychological Medicine, Institute of Psychiatry, Psychology and Neuroscience, King’s College London, London, UK; 2 School of Life Course & Population Sciences (SLCPS), King’s College London, London, UK; 3 Violence and Society Centre, University of London, London, UK; 4 National Centre for Social Research, London, UK; 5 Health Service and Population Research Department, IoPPN, King’s College London, London, UK; 6 Occupational Health, Safety and Wellbeing Service, Guy’s and St Thomas’ Hospitals NHS Foundation Trust, London, UK; 7 Department of Population Health Sciences, Faculty of Life Sciences and Medicine, King’s College London, London, UK; 8 Centre for Society and Mental Health, King’s College London, London, UK; 9 South London & Maudsley NHS Foundation Trust, London, UK

**Keywords:** Common Mental Disorders, epidemiology, health outcomes, population survey, social factors

## Abstract

**Aims:**

The associations of prior homelessness with current health are unknown. Using nationally representative data collected in private households in England, this study aimed to examine Common Mental Disorders (CMDs), physical health, alcohol/substance dependence, and multimorbidities in people who formerly experienced homelessness compared to people who never experienced homelessness.

**Methods:**

This cross-sectional study utilised data from the 2007 and 2014 Adult Psychiatric Morbidity Surveys. Former homelessness and current physical health problems were self-reported. Current CMDs, alcohol dependence and substance dependence were ascertained using structured validated scales. Survey-weighted logistic regression was used to compare multimorbidities (conditions in combination) for participants who formerly experienced homelessness with those who had never experienced homelessness, adjusting for sociodemographic characteristics, smoking status and adverse experiences. Population attributable fractions (PAFs) were calculated.

**Results:**

Of 13,859 people in the sample, 535 formerly experienced homelessness (3.6%, 95% CI 3.2–4.0). 44.8% of people who formerly experienced homelessness had CMDs (95% CI 40.2–49.5), compared to 15.0% (95% CI 14.3–15.7) for those who had never experienced homelessness. There were substantial associations between prior homelessness and physical multimorbidity (adjusted odds ratio [aOR] 1.98, 95% CI 1.53–2.57), CMD–physical multimorbidity (aOR 3.43, 95% CI 2.77–4.25), CMD–alcohol/substance multimorbidity (aOR 3.53, 95% CI 2.49–5.01) and trimorbidity (CMD–alcohol/substance–physical multimorbidity) (aOR 3.26, 95% CI 2.20–4.83), in models adjusting for sociodemographic characteristics and smoking. After further adjustment for adverse experiences, associations attenuated but persisted for physical multimorbidity (aOR 1.40, 95% CI 1.10–1.79) and CMD–physical multimorbidity (aOR 1.55, 95% CI 1.20–2.00). The largest PAFs were observed for CMD–alcohol/substance multimorbidity (17%) and trimorbidity (16%).

**Conclusions:**

Even in people currently rehoused, marked inequities across multimorbidities remained evident, highlighting the need for longer-term integrated support for people who have previously experienced homelessness.

## Introduction

Homelessness is an increasing social and public health emergency (World Health Organization, 2018). In the general population, the social gradient is considered a ‘slope’ of association between disadvantage and poor health outcomes (Marmot *et al.*, 1991). For homeless populations, health inequity is so severe that this has been conceptualised as a ‘cliff’ (Story, 2013). People who experience homelessness often experience multiple adversities (Bramley *et al.*, 2015), have high levels of secondary care service use (Field *et al.*, 2019) and premature mortality (Aldridge *et al.*, 2018).

There are evolving preferences around terminology for multiple health conditions. In policy, clinical practice, and research, multiple co-occurring health conditions are referred to as multimorbidity (Macmahon *et al.*, 2018). There are different types of multimorbidity, dependent on different combinations of conditions (Johnston *et al.*, 2019): for example, mental–physical multimorbidity includes co-occurring mental and physical conditions (Taloyan *et al.*, 2022). Studies of electronic health record data from patients accessing specialist homelessness primary care services have found high rates of multimorbidity and trimorbidity (co-occurring mental, physical and substance use conditions) (Bowen *et al.*, 2019; Queen *et al.*, 2017; Vallesi *et al.*, 2021). Similarly high rates of multimorbidity have been found in surveys of people in hostels, shelters, and homelessness outreach services (Rogans-Watson *et al.*, 2020; Vickery *et al.*, 2021). There are indications that rates of multimorbidity are increasing in homeless populations (Vickery *et al.*, 2021). People who have several diagnoses are more likely to be excluded from services, compounding barriers to accessing healthcare already faced by people who experience homelessness (Armstrong *et al.*, 2021).

Common Mental Disorders (CMDs) include types of depression and anxiety (Lewis *et al.*, 1992). A recent updated systematic review found that, despite a widespread acknowledgement that CMDs and multimorbidity are highly prevalent in homeless populations, studies have rarely assessed for comorbidities with mental disorders (Gutwinski *et al.*, 2021). There is evidence that people with concurrent mental and physical morbidities have worse health-related quality of life and clinical outcomes, as well as an increased risk of premature mortality, compared to those with physical or mental morbidities only (Macmahon *et al.*, 2018).

Concerns have been raised about the poor quality of data on people who have experienced homelessness (Kaushal *et al.*, 2021). Studies to date have largely focused on people who experience long-term homelessness and those who are in touch with specialist services. There is some research on formerly homeless populations living in settings such as supported accommodation and Housing First, however very little is known about health outcomes for people who move into private housing after homelessness (Demakakos *et al.*, 2020). Studies which have examined former homelessness in private households have largely focused on other outcomes, such as adverse experiences, mortality, and overlaps between homelessness and other domains of disadvantage and exclusion (Bramley and Fitzpatrick, 2018; Demakakos *et al.*, 2020; Sosenko *et al.*, 2020; Toro *et al.*, 2007). This is with the exception of two studies in the United States which have examined mental and physical health outcomes for people who previously experienced homelessness using private household survey data (Greenberg and Rosenheck, 2010) and electronic health records (Harper Sutherland and Rosenoff, 2021). Both studies found marked inequalities, particularly with mental health and substance use conditions, for people who had previously experienced homelessness.

There is a dearth of evidence on health outcomes for people who have previously experienced homelessness in England. We undertook a study using nationally representative data from people living in private households in England to assess the association of prior experiences of homelessness and co-occurring CMDs, physical health problems and alcohol/substance dependence (multimorbidities). Given evidence which has shown mental and physical health inequalities for currently homeless populations (Aldridge *et al.*, 2018; Gutwinski *et al.*, 2021), we hypothesized that we would observe a higher prevalence of these health outcomes for people who formerly experienced homelessness compared to those who never experienced homelessness.

## Methods

### Participants

The Adult Psychiatric Morbidity Survey (APMS) is a nationally representative stratified and multi-stage probability survey of adults (aged ≥16 years) living in private households in England. Private households are defined as owned and rented accommodation, including both private and social rented accommodation. The present study utilises data from the 2007 and 2014 waves of the survey (N = 14,949), from the first phases of survey data collection, which were conducted face-to-face by lay interviewers. Further details on the survey methods have been described elsewhere (Mcmanus *et al.*, 2020).

### Measures

To identify a prior experience of homelessness, the interviewer presented the participant with a card which included eight problems or events (including ‘being homeless’) and asked: ‘Now looking at this card, could you tell me if you have ever experienced any of these problems or events, at any time in your life?’. If a participant indicated that they had experienced ‘being homeless’ from this list, they were asked when they most recently experienced being homeless: within the last 6 months, over 6 months ago and after the age of 16, or over 6 months ago and before the age of 16.

The presence of current CMDs were ascertained using the Clinical Interview Schedule Revised (CIS-R) scale (Lewis *et al.*, 1992). The CIS-R is a structured validated instrument with high sensitivity and specificity to International Classification for Diseases 10 (ICD-10) CMD diagnoses (Lewis *et al.*, 1992). Lay interviewers ask about CMD symptoms over the last month, and then ask more detailed questions about symptoms in the previous week. A CIS-R score of ≥12 indicates presence of a CMD, and a score of ≥18 indicates severe symptoms (more symptoms and/or a longer duration of symptoms) which would likely warrant intervention (Stansfeld *et al.*, 2016). Participants’ responses to the CIS-R were mapped to diagnostic categories for CMDs (Stansfeld *et al.*, 2016), including depression (sub-categorised into mild depression, moderate depression, severe depression and combined depression groups), generalised anxiety disorder (GAD), panic disorder, phobias, obsessive-compulsive disorder, and CMD not otherwise specified (CMD-NOS). Participants who scored ≥12 on the CIS-R but did not meet the criteria for any specific CMD were included in the CMD-NOS group (Stansfeld *et al.*, 2016).

Participants were shown a list of physical health conditions or problems and reported if they had experienced these in the last 12-months. We included physical health conditions listed in both the 2007 and 2014 surveys (a total of 21 conditions, see Table 3).

Participants were asked to self-report their use of alcohol and illicit substances using a Computer-Assisted Self-Completion Interview. This section of the survey included the Alcohol Use Disorders Identification Test (AUDIT) (Saunders *et al.*, 1993) to assess harmful alcohol use. A score of 8–15 indicated hazardous drinking, 16–19 indicated harmful drinking and/or mild dependence and ≥20 indicated probable dependence (Room *et al.*, 2005). Substance dependence was ascertained by five questions designed to assess symptoms of drug dependence according to the Diagnostic Interview Schedule (including cannabis; amphetamines; cocaine; crack; ecstasy; heroin, methadone or physeptone; tranquillisers; glue, solvents, gas or aerosols) (McManus *et al.,* 2016). These questions asked about the past month and year and covered: daily use for 2 weeks or more, sense of need or dependence, inability to abstain, increased tolerance and withdrawal symptoms. A positive indication on any of these items was considered as an indication of possible dependence (McManus *et al.,* 2016).

### Outcomes

The primary outcome of this study was CMD–physical multimorbidity – defined as the co-occurrence of a CMD (ascertained by a score of ≥12 on the CIS-R) and one or more physical health problems (self-reported). We also ascertained the following binary health outcomes and multimorbidity types (Johnston *et al.*, 2019):

- CMDs (CIS-R ≥ 12).
- Physical health problem (≥1).
- Alcohol dependence (AUDIT ≥ 16).
- Substance dependence (≥1 possible dependence symptoms).
- Physical multimorbidity (≥2 physical health problems).
- CMD–substance multimorbidity (CMD and alcohol dependence and/or substance dependence).
- Trimorbidity (CMD, ≥1 physical health condition or problem and an alcohol problem (AUDIT score ≥16) and/or substance use dependence) (Hewett and Halligan, 2010).

### Covariates

The following sociodemographic variables were self-reported by participants: age (grouped into 10-year bands, with the exceptions of 16–34 and ≥55 due to small sample sizes), sex, ethnicity, marital status, employment status, educational attainment, and current housing tenure. Due to small sample sizes in the formerly homeless group, we aggregated Black ethnic groups (including Black, African, Caribbean and Black British groups), Asian ethnic groups (including Asian and Asian British) and Mixed/Multiple/other ethnic groups. For the same reason, we defined marital status as follows: those who were married, cohabiting, or in a same-sex couple were placed in one category, while those who were divorced, separated, or widowed were placed in another category. We were also interested in other adverse childhood and life-course experiences self-reported by participants in both 2007 and 2014 surveys, including problematic debt, problems with the police involving court appearances, violence in the home, sexual abuse, bullying, running away from home, spending time in an institution in childhood, and being expelled from school.

### Statistical methods

The analysis protocol was pre-registered on Open Science Framework (https://osf.io/sfyrq/). Stata version-17 was used (Statacorp, 2021), and survey weights for the APMS 2007 and 2014 combined surveys were implemented across all analyses using the *svy* command. Missing data for included variables were examined. Participants who had data missing for the homelessness, sociodemographic, or health variables were excluded, and a complete case analysis was undertaken. Descriptive characteristics of the combined sample were assessed, including raw frequencies and survey-weighted proportions for experiences of the last episode of homelessness, as well as sociodemographic characteristics, adverse experiences, health conditions/problems and multimorbidity by former experiences of homelessness.

Across all analyses, the exposure was a previous experience of homelessness. Multiple separate unadjusted logistic regression models were used to assess associations between prior experiences of homelessness and the health and multimorbidity outcomes. These models were then adjusted for age, sex, ethnicity, marital status, and survey year. Models were additionally adjusted for employment status and educational attainment, as individual-level indicators of socioeconomic position. A third model included additional adjustment for current smoking status. As adverse experiences were identified as potential confounders (Bramley and Fitzpatrick, 2018; Demakakos *et al.*, 2020), we additionally adjusted for childhood and life-course adverse experiences across multivariable logistic regression models. Confounders were selected a priori. Across all models, interactions by survey year and sex were assessed. As no interactions were evident, these variables remained in the models as putative confounders. Population attributable fractions (PAFs) for adjusted associations between prior homelessness and health outcomes were estimated using the STATA command *punaf* from the odds ratios obtained from weighted adjusted logistic regression models. PAFs estimate the proportion of outcome (e.g., CMD) risk in the population attributable to homelessness according to the derived model (Levine, 2007).

### Public and patient involvement

Nine people who had lived experience of homelessness were consulted throughout the research process for this study. Three people who advised on the study were connected with Rethink Mental Illness, a charity that works with people severely impacted by mental illness; six people were members of the Pathway Lived Experience Group, which is a homelessness healthcare charity. Advisors provided feedback at the study design stage to inform analyses – for example, their feedback on the analysis plan led to us distinguishing between CMD–physical multimorbidity and trimorbidity outcomes. People with lived experience were also consulted on which adverse experiences should be included as relevant confounders. Results were discussed with the two groups, and these discussions contributed towards interpretations of the data which have been highlighted in this paper.

### Role of the funding source

The funders were not involved in any aspect of the study design; in the collection, analysis and interpretation of data; in the writing of the report, or in the decision to submit this paper for publication.

## Results

**Figure 1.**
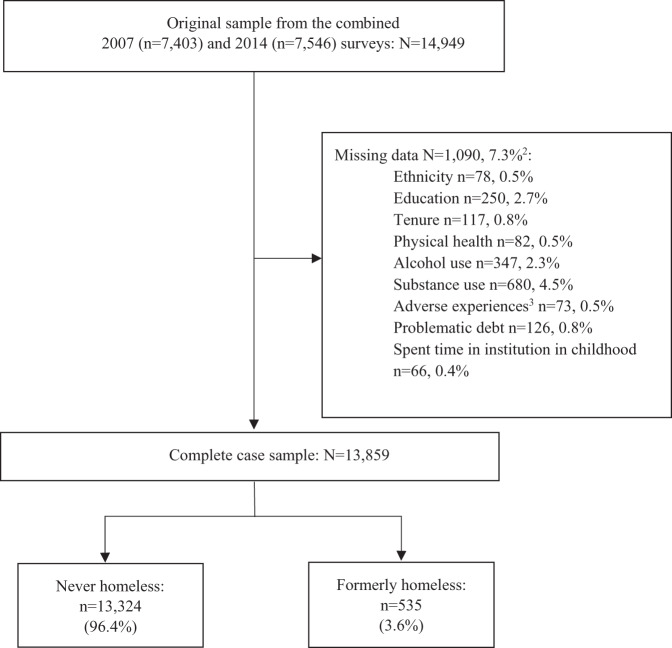
STROBE flow diagram.

^1^Proportions are unweighted. ^2^Groups for missing data are not mutually exclusive. ^3^Adverse experiences include the following variables: homelessness, problem with police involving court appearance, violence in the home, sexual abuse, bullying, expelled from school, running away from home.

Figure 1 shows how the sample was derived. Missing data was low (only 7.3% across the sample, more detail in Appendix 1). Out of the complete 2007 and 2014 combined case sample (n = 13,859), 535 participants reported a previous experience of homelessness. The prevalence estimate for at least one prior experience of homelessness was 3.6% (95% CI 3.3–4.0). Out of those who had experienced homelessness, 444 (82.0%) last experienced homelessness more than 6 months ago aged 16 or older, 76 (14.9%) last experienced homelessness more than 6 months ago before the age of 16 and 15 (3.1%) experienced homelessness in the 6 months before the survey interview.

Table 1 shows the sociodemographic characteristics of the sample and adverse experiences, by prior experiences of homelessness. Men and women were evenly split in both the formerly homeless and never homeless groups. In total, 69.2% of people who had never experienced homelessness owned their current property; conversely, 68.3% of the formerly homeless group lived in rented accommodation, of which the majority lived in social housing (47.0%). People who had experienced homelessness had experienced more adverse childhood and life-course experiences, compared to people who had never experienced homelessness, with large differences observed between groups. Over half of the formerly homeless group (52.2%) had experienced bullying, and over a third (40.0%) had experienced violence in their homes.

**Table 1.** Sociodemographic characteristics and adverse experiences of respondents to the Adult Psychiatric Morbidity Surveys, by experience of homelessness^a^

	Never homeless (N = 13,324)			Formerly homeless (N = 535)			
	N	%	95% CI	N	%	95% CI	Total N
Combined sample	13324	96.4	96.0−96.7	535	3.6	3.3−4.0	13859
Age, years							
16−34	2343	16.3	15.6−17.0	121	21.2	17.6−25.2	2464
35−44	2173	17.5	16.8−18.3	112	22.3	18.5−26.6	2285
45−54	5962	35.2	34.3−36.1	130	18.6	15.4−22.3	6092
≥55	2846	31.0	29.9−32.0	172	38.0	33.2−42.9	3018
Sex^b^							
Male	5529	48.5	47.5−49.5	228	50.9	46.1−55.6	5757
Female	7795	51.5	50.5−52.5	307	49.1	44.4−53.9	8102
Ethnicity							
White British	11612	83.4	82.4−84.4	435	76.6	71.7−80.9	12047
White other	649	5.8	5.3−6.3	22	5.8	3.6−9.2	671
Black/African/Caribbean/Black British	306	2.7	2.4−3.2	42	9.9	6.9−13.8	348
Asian/Asian British	493	5.5	4.8−6.2	15	2.5	1.4−4.4	508
Mixed/Multiple/Other ethnic groups	264	2.6	2.2−3.0	21	5.3	3.1−8.7	285
Marital status							
Married/cohabiting/same sex couple	7603	63.3	62.3−64.3	189	48.6	43.9−53.4	7792
Single	2592	23.0	22.0−24.0	182	30.0	25.9−34.5	2774
Divorced/separated/widowed	3129	13.7	13.2−14.3	164	21.3	18.0−25.1	3293
Employment status							
In employment	7365	61	60.0−61.9	249	51.0	46.1−55.8	7614
Unemployed	307	2.8	2.4−3.2	39	8.1	5.6−11.5	346
Economically inactive	5652	36.3	35.3−37.2	247	40.9	36.2−45.8	5899
Educational attainment^c^							
Degree	4011	31.0	30.1−32.0	128	23.7	19.9−28.0	4139
A Level	1966	17.0	16.2−17.8	85	18.7	15.2−22.9	2051
GCSE or equivalent	3250	26.0	25.2−26.9	154	29.0	24.8−33.6	3404
Foreign/other	498	3.3	3.0−3.6	13	2.1	1.2−3.9	511
No qualifications	3599	22.7	21.9−23.5	155	26.4	22.3−30.9	3754
Tenure							
Owner-occupier	9367	69.2	68.1−70.2	159	31.7	27.4−36.4	9526
Social renter	2087	14.3	13.6−15.2	268	47.0	42.0−51.9	2355
Private or other renter	1870	16.5	15.6−17.4	108	21.3	17.6−25.6	1978
Adverse experiences^d^							
Spent time in institution (up until age 16)	288	2.0	1.7−2.2	68	13.8	10.7−17.7	356
Expelled from school	193	1.8	1.5−2.1	70	15.3	11.9−19.6	263
Ran away from home	483	3.7	3.3−4.1	175	33.9	29.6−38.5	658
Bullying	2736	21.2	20.4−22.1	285	52.2	47.3−57.1	3021
Violence in home	1047	6.8	6.3−7.2	231	40.0	35.4−44.8	1278
Sexual abuse	647	4.2	3.9−4.6	137	23.5	19.8−27.7	784
Problem with police involving court appearance	753	5.9	5.4−6.4	148	29.6	25.2−34.3	901
Problem debt (in past year)	898	6.7	6.2−7.2	180	32.2	27.8−36.9	1078
	**Mean**	**SE**	**95% CI**	**Mean**	**SE**	**95% CI**	–
Average number of adverse experiences	0.52	0.01	0.50−0.54	2.41	0.09	2.24−2.57	–

a Data are shown as raw sample n, survey weighted proportions % (with 95% Confidence Intervals). Pearson’s χ^2^-test *p*-values < 0.0001, unless indicated otherwise.

b *p* = 0.338.

c *p* = 0.011.

d At any time in life, unless indicated otherwise.

Table 2 shows the frequencies and prevalence estimates for health conditions and multimorbidities, by prior experiences of homelessness. The majority of both the formerly homeless and never homeless groups self-reported at least one physical health condition or problem. Higher prevalence estimates were observed for the formerly homeless group for the following physical health conditions: migraines or frequent headaches, asthma, allergies, stomach ulcer/digestive problems, liver problems, back/bone/joint/muscle problems, infectious disease and skin problems.

**Table 2.** Survey-weighted prevalence estimates for health conditions and problems, by experience of homelessness

	Never homeless (N = 13,324)			Formerly homeless (N = 535)			
	N	%	95% CI	N	%	95% CI	Pearson’s χ^2^-test *p*-values
Common Mental Disorders (CMD)							
Moderate (CIS-R^a^ score 12−17)	986	7.2	6.7−7.8	90	6.5	6.1−7.0	<0.0001
Severe (CIS-R score 18+)	940	14.1	11.5−17.3	165	29.7	25.4−34.3	
Physical health^b^							
≥1 Physical health condition	9516	67.3	66.2−68.3	422	76.6	72.0−80.6	0.0002
Cancer	242	1.4	1.3−1.6	10	1.3	0.4−2.1	0.70
Diabetes	794	5.2	4.8−5.6	31	5.2	3.0−7.4	0.90
Epilepsy/fits	70	0.5	0.4−0.6	10	1.4	0.5−2.3	0.004
Migraine or frequent headaches	1779	13.0	12.3−13.7	120	22.5	18.3−26.7	<0.0001
Cataracts/eyesight problems	2284	15.4	14.6−16.2	97	16.0	12.9−19.1	0.69
Ear/hearing problems	1471	9.5	9.0−10.1	49	8.0	5.4−10.7	0.30
Heart attack/angina	339	2.1	1.8−2.3	12	2.2	0.6−3.7	0.89
High blood pressure	2708	17.0	16.3−17.7	85	17.4	13.5−21.3	0.86
Bronchitis/emphysema	353	2.2	1.9−2.4	19	2.8	1.4−4.1	0.34
Asthma	1203	8.5	8.0−9.1	81	13.5	10.6−16.5	0.0001
Allergies	1396	10.6	9.9−11.2	90	16.7	13.1−20.2	0.0001
Stomach ulcer/digestive problems	968	6.6	6.1−7.0	56	10.3	7.3−13.3	0.003
Liver problems	108	0.7	0.6−0.9	21	3.1	1.7−4.5	<0.0001
Bowel/colon problems	849	5.7	5.2−6.0	44	8.1	5.6−10.7	0.03
Bladder problems/incontinence	678	4.1	3.7−4.4	37	5.9	3.8−7.9	0.04
Arthritis	2375	14.2	13.6−14.8	93	13.9	10.9−16.9	0.85
Bone/back/joint or muscle problems	3601	25.1	24.3−26.0	191	32.6	28.0−37.3	0.001
Infectious disease	51	0.4	0.3−0.5	12	2.4	0.9−3.9	<0.0001
Skin problems	1452	10.6	10.0−11.2	94	19.3	15.3−23.4	<0.0001
Alcohol Problem (AUDIT^c^ score)							
Hazardous drinking (8−15)	2064	17.6	16.8−18.4	95	19.9	16.2−24.2	<0.0001
Harmful drinking/mild dependence (16−19)	215	1.8	1.5−2.1	14	2.2	1.2−4.0	
Probable dependence (≥20)	143	1.2	1.0−1.5	28	5.1	3.4−7.7	
Substance dependence	269	2.7	2.3−3.1	73	15.1	11.8−19.1	<0.0001
Multimorbidity							
Physical multimorbidity^d^	6021	40.2	39.2−41.2	303	54.0	49.0−58.9	<0.0001
CMD–physical multimorbidity^e^	1763	12.1	11.5−12.8	225	37.7	33.2−42.5	<0.0001
CMD–alcohol/substance multimorbidity^f^	218	1.7	1.5−2.0	69	12.2	9.4−15.8	<0.0001
Trimorbidity^g^	176	1.3	1.1−1.6	55	8.9	6.6−11.9	<0.0001

a Clinial Interview Schedule-Revised.

b Estimates for dementia or Alzheimer’s disease and stroke not presented due to small cell counts (n < 5 for formerly homeless group).

c Alcohol Use Disorders Identification Test- groups are mutually exclusive.

d Physical multimorbidity: ≥2 physical health conditions/problems.

e CMD–physical multimorbidity: CMD (CIS-R ≥12) & ≥1 physical health problem.

f CMD–substance multimorbidity: CMD (CIS-R ≥12) and alcohol dependence (AUDIT ≥16) and/or substance dependence.

g Trimorbidity: CMD (CIS-R ≥12) and ≥1 physical health problem & alcohol dependence (AUDIT ≥16) and/or substance dependence.

Almost half of the formerly homeless group met criteria for current CMDs (44.8%, 95% CI 40.2–49.5) compared to 15.0% of the never homeless group (95% CI 14.3–15.7). A high proportion of the formerly homeless group experienced severe CMD symptoms (29.7%, 95% CI 25.4–34.3) (Table 2). People who had experienced homelessness were more likely to screen positive for all types of CMDs, compared to those who never experienced homelessness (Appendix 2). Depressive disorders, GAD and CMD-NOS, appeared to account for most of the excess of CMDs. People who formerly experienced homelessness had almost six times the prevalence of current substance dependence compared to people who had never been homeless (Table 3). The formerly homeless group had over four times the prevalence of probable alcohol dependence (Table 3).

**Table 3.** Stepped adjusted logistic regression models examining associations between former homelessness and multimorbidities^a^

	N (13,859)		Unadjusted		Adjusted for demographic characteristics and survey year^b^		Additionally adjusted for socio-economic position^c^		Additionally adjusted for smoking^d^		Additionally adjusted for adverse experiences^e^		
	Never homeless (13,324)	Formerly homeless (535)	OR	95% CI	OR	95% CI	OR	95% CI	OR	95% CI	OR	95% CI	*p-value*
Common Mental Disorder (CMD)	2,099	259	4.61	3.77−5.62	4.28	3.50−5.24	3.88	3.18−4.74	3.45	2.81−4.23	1.54	1.21−1.97	0.001
Physical health problem	113	422	1.59	1.25−2.03	2.05	1.59−2.64	1.99	1.54−2.58	1.97	1.52−2.56	1.31	0.99−1.74	0.057
Alcohol dependence	493	42	2.57	1.76−3.75	2.22	1.51−3.27	2.20	1.48−3.27	1.61^j^	1.07−2.43	0.85	0.54−1.34	0.479
Substance dependence	462	73	6.47	4.66−8.97	5.73	4.04−8.12	5.04	3.53−7.19	3.10	2.14−4.51	1.55	1.01−2.37	0.046
Physical multimorbidity^f^	232	303	1.74	1.42−2.14	2.41	1.93−2.99	2.27	1.81−2.83	2.25	1.80−2.83	1.40	1.10−1.79	0.006
CMD–physical multimorbidity^g^	1,763	225	4.40	3.57−5.42	4.26	3.45−5.26	3.86	3.13−4.78	3.44	2.77−4.27	1.55	1.20−2.00	0.001
CMD–alcohol/substance multimorbidity^h^	218	69	7.89	5.64−11.03	6.33	4.51−8.88	5.13	3.65−7.21	3.54	2.49−5.04	1.35	0.89−2.04	0.153
Trimorbidity^i^	176	55	7.16	5.00−10.26	5.76	3.97−8.37	4.81	3.29−7.03	3.29	2.22−4.87	1.22	0.77−1.94	0.394

a All *p*-values <0.0001 except where otherwise indicated.

b Adjusted for age, sex, ethnicity, marital status, survey year.

c Adjusted for age, sex, ethnicity, marital status, survey year, educational attainment, employment status.

d Adjusted for age, sex, ethnicity, marital status, survey year, educational attainment, employment status, smoking status.

e Adjusted for age, sex, ethnicity, marital status, survey year, educational attainment, employment status, smoking status, debt in past year, problems with police involving court appearance, violence in the home, sexual abuse, bullying, living in an institution up until age 16, expelled from school, running away from home.

f Physical multimorbidity: ≥2 physical health conditions/problems.

g CMD–physical multimorbidity: Common Mental Disorder (CIS-R ≥12) and ≥1 physical health condition/problem.

h CMD–alcohol/substance multimorbidity: Common Mental Disorder (CIS-R ≥12) and alcohol dependence (AUDIT ≥16) and/or substance dependence.

i Trimorbidity: Common Mental Disorder (CIS-R ≥12) and ≥1 physical health condition/problem & alcohol dependence (AUDIT ≥16) and/or substance dependence.

j *p* = 0.024.

The formerly homeless group also had substantially higher prevalence estimates for all types of multimorbidity. The most common type of multimorbidity experienced by both formerly homeless and never homeless groups was physical multimorbidity, which was present in over half of the formerly homeless group (54.0%, 95% CI 49.0–58.9), compared to under half of those who had never experienced homelessness (40.2%, 95% CI 39.2–41.2). The largest absolute difference in prevalence between the formerly homeless and never homeless groups was observed for CMD–physical multimorbidity (formerly homeless = 37.7%, 95% CI 33.2–42.5; never homeless = 12.1%, 95% CI 11.5–12.8). Appendix 3 includes quasi-proportional Euler diagrams which illustrate the mutually exclusive overlaps between CMDs, physical and alcohol/substance dependence domains for the two groups. People who had formerly experienced homelessness rarely experienced alcohol/substance dependence or CMDs in isolation. The prevalence of trimorbidity was almost seven times higher for the formerly homeless group.

Table 3 includes the crude and adjusted odds ratios (aORs) and 95% confidence intervals for the logistic regression models examining the associations between former homelessness and multimorbidities. People who formerly experienced homelessness were more likely to experience all morbidities and multimorbidities. Particularly, people who were formerly homeless were over sevenfold more likely to experience CMD–alcohol/substance multimorbidity (OR 7.89, 95% CI 5.64–11.03) and trimorbidity (OR 7.16, 95% CI 5.00–10.26). Associations between prior experience of homelessness and all health outcomes persisted even after adjustment for demographic characteristics, survey year, indicators of socioeconomic position and smoking. The largest aORs were observed for CMD–alcohol/substance multimorbidity (aOR 3.54, 95% CI 2.49–5.04), CMDs (aOR 3.45, 95% CI 2.81–4.23) and CMD–physical multimorbidity (aOR 3.44, 95% CI 2.77–4.27).

In subsequent analyses, we further adjusted for other adverse experiences (Table 3). We found that these attenuated some of the associations with health outcomes to an extent. This was particularly the case for multimorbidities including substance or alcohol dependence, where associations were no longer evident. However, associations between homelessness and CMDs, physical health problems, physical multimorbidity and CMD–physical multimorbidity remained after adjustment for adverse experiences.

PAFs are presented in Table 4. While 3.6% of the private household population had experienced homelessness, the largest PAFs were observed for CMD–alcohol/substance multimorbidity (17%), trimorbidity (16%) and substance dependence (13%).

**Table 4.** Adjusted population attributable fractions for logistic regression models adjusted for age, sex, ethnicity, marital status, survey year, educational attainment, employment status, smoking status

	Never homeless (N=13,324)	Formerly homeless (N=535)	Adjusted population attributable fraction (%)	95% CI
Common Mental Disorders (CMD)	2,099	259	6	5−7
Physical health problem	113	422	1	1−1
Alcohol problem	493	42	4	2−7
Substance dependence	462	73	13	9−18
Physical multimorbidity^a^	232	303	2	1−2
CMD–physical multimorbidity^b^	1,763	225	7	6−8
CMD–alcohol/substance multimorbidity^c^	218	69	17	12−22
Trimorbidity^d^	176	55	16	10−21

a Physical multimorbidity: ≥2 physical health conditions/problems.

b CMD–physical multimorbidity: CMD (CIS-R ≥12) and ≥1 physical health condition/problem.

c CMD–substance/alcohol multimorbidity: CMD (CIS-R ≥12) and alcohol dependence (AUDIT ≥16) and/or substance dependence.

d Trimorbidity: CMD (CIS-R ≥12) and ≥1 physical health condition/problem and alcohol dependence (AUDIT ≥16) and/or substance dependence.

## Discussion

In a nationally representative sample of adults living in private households, people with a prior experience of homelessness had a substantially increased likelihood of CMDs, physical health problems, alcohol dependence, substance dependence, multimorbidity and trimorbidity. For people who experienced prior homelessness, the odds of CMD–physical multimorbidity were over fourfold compared with those who had never experienced homelessness. Adjusting for adverse life experiences fully attenuated associations with alcohol and substance dependence morbidities and multimorbidities (including trimorbidity), however associations remained evident for CMDs, physical multimorbidity and CMD–physical multimorbidity. Our estimates suggest that prior experiences of homelessness may have significant deleterious associations with adverse mental and physical health, which persist even after people have been re-housed, and after adjustment for other adverse experiences. Furthermore, despite homelessness affecting 3.6% of people in private households, 17% of mental-substance multimorbidity and 16% of trimorbidity were attributable to prior experiences of homelessness in a nationally representative sample. This indicates the public health significance of preventing homelessness for multimorbidities.

Our study complements and extends the inclusion health literature in several ways. To our knowledge, no other study has assessed the prevalence of CMDs and multimorbidity using nationally representative data from people in private households in England who have formerly experienced homelessness. A systematic review and meta-analysis found a 12.6% prevalence of major depression in currently homeless populations (Gutwinski *et al.*, 2021). Similarly, we found a 13.2% prevalence of depression for people who had formerly experienced homelessness. Taken together, this evidence indicates that an elevated prevalence of CMDs persist beyond the initial experience of homelessness, even when people have moved into private housing and after adjusting for adverse experiences. We also found that people who previously experienced homelessness were more likely to experience more severe symptoms of CMDs, which indicates a higher likelihood of warranting treatment. Previous research has found that more severe CMD symptoms are associated with a worse quality of life, higher rates of unemployment, more days off sick and higher suicidality (Das-Munshi *et al.*, 2008). Furthermore, a recent systematic review and meta-analysis has found that housing status is an important determinant of depression treatment prognosis (Buckman *et al.*, 2022). The granular detail on CMD severity in the present study provides novel insights into the mental health of people following their experience of homelessness.

The prevalence of self-reported physical health problems reflected patterns found in previous research with people currently experiencing homelessness. For example, we found a higher prevalence of asthma among people who had previously experienced homelessness. This has similarly been found in a previous study with people who were rough sleeping in England (Lewer *et al.*, 2019). Participants who formerly experienced homelessness had a lower prevalence of diabetes compared to never homeless participants, which has also been found in previous studies of rough sleeper populations (Bernstein *et al.*, 2015; Lewer *et al.*, 2019). It has been suggested that this could be due to under-diagnosis (Bernstein *et al.*, 2015).

We found a higher prevalence of alcohol and substance dependence in the formerly homeless group, which is consistent with other studies for currently homeless populations (Gutwinski *et al.*, 2021). We found that associations with alcohol and substance dependence fully attenuated after adjusting for other life-course adverse experiences, indicating a possible relationship between multiple disadvantage and substance use. Our PAF analysis further highlights the potential contribution of prior homelessness to alcohol and substance dependence and multimorbidities.

Women are often under-represented in homelessness research. In the present study, the formerly homeless group was evenly split by sex. This contrasts with previous research on multimorbidity and homelessness in the United Kingdom, which focus on sub-populations of homelessness in specialist services where men make up >75% of samples (Bowen *et al.*, 2019; Queen *et al.*, 2017; Rogans-Watson *et al.*, 2020). Our study therefore extends the evidence-base by including a higher proportion of women who have experienced homelessness. This study addresses an important gap in the literature by assessing health inequalities in multimorbidity for people who have previously experienced homelessness. A key strength of this dataset is that it is nationally representative, and therefore generalisable to people living in private households in England. The APMS does not include the same biases as healthcare service use datasets, as the latter only includes people in contact with health services, which has tended to form the basis of most previous research in this area. By using structured validated instruments, we could identify potentially undiagnosed and untreated CMDs and alcohol/substance dependence (Mcmanus *et al.*, 2020). The self-report module for alcohol and substance use disorders may have enabled participants to report current use without feeling stigmatised, as these questions were asked via a computer rather than face-to-face with an interviewer (Byron *et al.*, 2016). Our study design enabled comparisons with people who had never experienced homelessness, while much of the previous work in this area have lacked population controls. We were also able to pool samples over consecutive years of survey, which enhanced study power to be able to detect differences. Our modelling approaches included a range of putative confounders, including socioeconomic position indicators, smoking and adverse experiences, which have not been adjusted for in previous studies. We involved people with lived experience of homelessness in the research process, which led to changes in the analyses including investigating trimorbidity as an outcome and the selection of relevant confounders. The research process was therefore enhanced and enriched by people who the research applies to.

However, our study has several limitations. First, our analyses relied on cross-sectional data, which limits our ability to draw conclusions about causality. Although we looked at previous experiences of homelessness and current health conditions, we cannot make inferences on the temporality between homelessness and health using this data. There is some evidence that mental health and substance use problems often pre-date homelessness (Fitzpatrick *et al.*, 2013), and the relationship between health conditions and homelessness is complex and likely to be bidirectional. While we were able to adjust for multiple potential confounders, there may have also been unmeasured confounders in the present observational study. We did not have data on the duration and type of homelessness experienced by participants. The broad definition of homelessness means that the generalizability of this research to people with specific experiences (e.g., temporary accommodation) is unclear. Furthermore, the time between experiencing homelessness and becoming housed may be highly variable between individuals. It is a limitation that these desirable details on experiences of homelessness were not included in the survey. Furthermore, while education and employment are indicative of social opportunities and income (Galobardes *et al.*, 2007), these variables are unlikely to provide a complete reflection of socioeconomic status on their own. Other socioeconomic indicators which were not included may account for some of these associations between homelessness and health outcomes, such as financial circumstances including direct measures of income and wealth (Galobardes *et al.*, 2007; Shavers, 2007).

Information which is self-reported, including homelessness, may be subject to social desirability bias, and as such may have been under-reported in our sample. It is a limitation that homelessness was not defined during data collection. Some people may not have considered themselves to have experienced homelessness and may not have self-identified, despite experiences which would meet the criteria under homelessness frameworks and definitions (e.g., overcrowding) (Edgar, 2012). However, our 3.6% estimated prevalence of homelessness is comparable to other private household survey findings from the United Kingdom, where estimates of former homelessness range between 1.6% and 7.7% (Demakakos *et al.*, 2020, Bramley and Fitzpatrick, 2018, Toro *et al.*, 2007).

The health conditions included in our study are not exhaustive, for example this does not include severe mental health conditions. Furthermore, our measure for physical health was based on self-report. It is possible that in our study this led to an under-reporting of some physical health conditions/problems (e.g., for diabetes and high blood pressure), particularly if not known to the participant or if managed without medications. Despite this, our prevalence estimates for physical health conditions are broadly comparable to other studies which have used clinician diagnoses or other methods to ascertain physical health (Aldridge *et al.*, 2018), suggesting that this was not a particularly prominent source of bias in our study. People who had missing data differed from people who had complete data by sociodemographic characteristics and health outcomes (Appendix 1); however, due to the low levels of missingness, this was unlikely to have impacted estimates.

Lastly, this APMS study used data from surveys in 2007 and 2014. There is some evidence to suggest that inequalities in health outcomes for people who experience homelessness may have widened since 2014 (Vickery *et al.*, 2021), and conditions of homelessness may also have changed over time. Therefore, the prevalence estimates and associations in the APMS data may be an underestimate compared to present-day estimates.

In conclusion, this research shows that prior experiences of homelessness have associations for adverse mental health, physical health, and alcohol/substance dependence, even after people have been rehoused in private households. This could account for stark reductions in life expectancy known to impact this group (Demakakos *et al.*, 2020). While policies and clinical practice guidelines are largely focused on currently homeless populations, the present study highlights that there is a need for longer-term support for people after their experience of homelessness. The National Institute for Health and Clinical Excellence (NICE) guidelines for treating people who experience homelessness advises trauma-informed approaches to care (Nice, 2022). Given the findings on the high prevalence of depression, anxiety, and adverse experiences in the formerly homeless group, the present research indicates the potential value of applying these recommendations for trauma-informed care for people post-homelessness. Interventions which address alcohol and substance-related multimorbidity and address multiple adversities could have the biggest impact on population health. The findings on multimorbidity demonstrate the need for the integration, collaboration and co-ordination between multiple health and social care services to support this population. In clinical practice, assessments and recordings of prior homelessness are likely to be beneficial. Lastly, the findings from this study underscore the value of the life-course prevention of homelessness to reduce health inequalities.

## Supporting information

Chilman et al. supplementary material Chilman et al. supplementary material

## Data Availability

The dataset was de-identified prior to researcher access. The 2007 dataset is open access. The 2014 APMS dataset was available under a Special License, which required a Data Sharing Agreement (DSA) with NHS Digital. A Data Access Request for the present study was approved by NHS Digital in 2019 and extended in 2023 (reference: DARS-NIC-320217-X8P0W-V0.3). The data are held securely on the King’s Centre for Military Health Research (KCMHR) server, which is connected to the department where this research took place (Department of Psychological Medicine), complying with a KCMHR NHS Data Security Protection Toolkit.

## Supplementary material

The supplementary material for this article can be found at https://doi.org/10.1017/S2045796024000659.
