# Supplementary material for: The public health significance of prior homelessness: findings on multimorbidity and mental health from a nationally representative survey
Source: Epidemiol Psychiatr Sci. 2024 Nov 8;33:e63. doi: 10.1017/S2045796024000659 (PMC11669814; doi:10.1017/S2045796024000659)
Supplement: Chilman et al. supplementary material [file S2045796024000659sup001.docx]

Contents

[1. Characteristics of participants with/without missing data. 2](#_Toc170301479)

[2. Prevalence of ICD-10 Common Mental Disorders (CMDs), by experiences of prior homelessness. * 5](#_Toc170301480)

[3. Quasi-proportional Euler diagrams illustrating overlaps between health problems for the formerly homeless group and never homeless group*. 6](#_Toc170301481)

[4. STROBE checklist for observational studies. 7](#_Toc170301482)

[5. Appendix references 12](#_Toc170301483)

# 1. Characteristics of participants with/without missing data.

|  | Participants with no missing data (complete case sample) (N=13859, 92.7%) | | Participants with missing data (N=1090, 7.3%) | | Pearsons *X^2^* test *p-value* |
| --- | --- | --- | --- | --- | --- |
|  | **N** | **%^1^** | **N** | **%^1^** |  |
| Homelessness | |  |  |  |  |
| Never homeless | 13324 | 96.1 | 953 | 93.7 | <0.0001 |
| Formerly homeless | 535 | 3.9 | 64 | 6.3 |  |
|  |  |  |  |  |  |
| Age |  |  |  |  |  |
| 16-34 | 3018 | 21.8 | 180 | 16.5 | <0.0001 |
| 35-44 | 2464 | 17.8 | 129 | 11.8 |  |
| 45-54 | 2285 | 16.5 | 139 | 12.8 |  |
| 55+ | 6092 | 44.0 | 642 | 58.9 |  |
|  |  |  |  |  |  |
| Sex |  |  |  |  |  |
| Male | 5757 | 41.5 | 498 | 45.7 | 0.008 |
| Female | 8102 | 58.5 | 592 | 54.3 |  |
|  |  |  |  |  |  |
| Ethnicity |  |  |  |  | 0.008 |
| White British | 12047 | 86.9 | 839 | 82.9 |  |
| White Other | 671 | 4.8 | 63 | 6.2 |  |
| Black / African / Caribbean / Black British | 348 | 2.5 | 37 | 3.7 |  |
| Asian / Asian British | 508 | 3.7 | 48 | 4.7 |  |
| Mixed /Multiple ethnic groups/ Other ethnic groups | 285 | 2.1 | 25 | 2.5 |  |
|  |  |  |  |  |  |
| Marital status | |  |  |  | <0.0001 |
| Married/cohabiting/same sex couple | 7792 | 56.2 | 478 | 43.9 |  |
| Single | 2774 | 20.0 | 242 | 22.2 |  |
| Divorced/separated/widowed | 3293 | 23.8 | 370 | 33.9 |  |
|  |  |  |  |  |  |
| Employment status | |  |  |  | <0.0001 |
| In employment | 7614 | 54.9 | 371 | 34.0 |  |
| Unemployed | 346 | 2.5 | 36 | 3.3 |  |
| Economically inactive | 5899 | 42.6 | 683 | 62.7 |  |
|  |  |  |  |  |  |
| Educational attainment | | |  |  | <0.0001 |
| Degree | 4139 | 29.9 | 187 | 22.3 |  |
| A Level | 2051 | 14.8 | 79 | 9.4 |  |
| GCSE or equivalent | 3404 | 24.6 | 160 | 19 |  |
| Foreign/other | 511 | 3.7 | 47 | 5.6 |  |
| No qualifications | 3754 | 27.1 | 367 | 43.7 |  |
|  |  |  |  |  |  |
| Tenure |  |  |  |  | <0.0001 |
| Owner-occupier | 9526 | 68.7 | 569 | 58.5 |  |
| Social renter | 2355 | 17.0 | 272 | 28.0 |  |
| Private or other renter | 1978 | 14.3 | 132 | 13.6 |  |
|  |  |  |  |  |  |
| Problem debt in past year | |  |  |  | <0.0001 |
| No | 12781 | 92.2 | 879 | 91.2 |  |
| Yes | 1078 | 7.8 | 85 | 8.8 |  |
|  |  |  |  |  |  |
| Problem with police involving court appearance | | | | | <0.0001 |
| Not mentioned | 12958 | 93.5 | 914 | 89.9 |  |
| Mentioned | 901 | 6.5 | 103 | 10.1 |  |
|  |  |  |  |  |  |
| Violence in home | |  |  |  | 0.737 |
| Not mentioned | 12581 | 90.8 | 920 | 90.5 |  |
| Mentioned | 1278 | 9.2 | 97 | 9.5 |  |
|  |  |  |  |  |  |
| Sexual abuse at any time in life | | | |  | 0.643 |
| Not mentioned | 13075 | 94.3 | 963 | 94.7 |  |
| Mentioned | 784 | 5.7 | 54 | 5.3 |  |
|  |  |  |  |  |  |
| Bullying at any time in life | | |  |  | 0.315 |
| Not mentioned | 10838 | 78.2 | 809 | 79.5 |  |
| Mentioned | 3021 | 21.8 | 208 | 20.5 |  |
|  |  |  |  |  |  |
| Spent time in institution up to age 16 | | | |  | 0.146 |
| No | 13503 | 97.4 | 990 | 96.7 |  |
| Yes | 356 | 2.6 | 34 | 3.3 |  |
|  |  |  |  |  |  |
| Expelled from school | | |  |  | <0.0001 |
| No | 13596 | 98.1 | 981 | 96.5 |  |
| Yes | 263 | 1.9 | 36 | 3.5 |  |
|  |  |  |  |  |  |
| Running away from home | | |  |  | 0.598 |
| No | 13201 | 95.3 | 965 | 94.9 |  |
| Yes | 658 | 4.7 | 52 | 5.1 |  |
|  |  |  |  |  |  |
| Common Mental Disorder | |  |  |  | <0.0001 |
| Not present | 11501 | 83.0 | 834 | 76.5 |  |
| Present | 2358 | 17.0 | 256 | 23.5 |  |
|  |  |  |  |  |  |
| 1+ physical health problem | | |  |  | 0.012 |
| Not present | 3921 | 28.3 | 248 | 24.6 |  |
| Present | 9938 | 71.7 | 760 | 75.4 |  |
|  |  |  |  |  |  |
| Alcohol dependence |  |  |  |  | 0.008 |
| No dependence (AUIDT score <16) | 13459 | 97.1 | 709 | 95.4 |  |
| Dependence (AUDIT score ≥16) | 400 | 2.9 | 34 | 4.6 |  |
|  |  |  |  |  |  |
| Substance dependence | |  |  |  | <0.0001 |
| Dependent on cannabis only | 247 | 1.8 | 12 | 2.9 |  |
| Dependent on any other drug (with or without cannabis) | 95 | 0.7 | 9 | 2.2 |  |
| No dependency | 13517 | 97.5 | 389 | 94.9 |  |
|  |  |  |  |  |  |
| Physical multimorbidity | | |  |  | <0.0001 |
| No multimorbidity | 7535 | 54.4 | 473 | 46.9 |  |
| Multiple physical conditions/problems | 6324 | 45.6 | 535 | 53.1 |  |
|  |  |  |  |  |  |
| Mental-physical multimorbidity | | |  |  | <0.0001 |
| No multimorbidity | 11871 | 85.7 | 876 | 80.4 |  |
| CMD and physical health | 1988 | 14.3 | 214 | 19.6 |  |
|  |  |  |  |  |  |
| Mental-substance multimorbidity |  |  |  |  | 0.133 |
| No multimorbidity | 13572 | 97.9 | 1060 | 97.2 |  |
| CMD and alcohol/substance dependency | 287 | 2.1 | 30 | 2.8 |  |
|  |  |  |  |  |  |
| Trimorbidity | | |  |  | 0.013 |
| No trimorbidity | 13628 | 98.3 | 302 | 96.5 |  |
| Trimorbidity | 231 | 1.7 | 11 | 3.5 |  |
| ^1^Proportions are not survey weighted. | | | | | |

2. Prevalence of ICD-10 Common Mental Disorders (CMDs), by experiences of prior homelessness. *

|  | Never homeless  (N=13,324) | | | Formerly homeless  (N=535) | | |
| --- | --- | --- | --- | --- | --- | --- |
|  | **N** | **%** | **95% CI** | **N** | **%** | **95% CI** |
| Mild depression^1^ | 147 | 1.0 | 0.8-1.2 | 12 | 2.3 | 1.3-4.1 |
| Moderate depression | 244 | 1.6 | 1.4-1.8 | 63 | 10.9 | 8.3-14.2 |
| Severe depression (f32.2^2^) | 130 | 0.8 | 0.6-1.0 | 36 | 6.2 | 4.3-9.0 |
| Depressive episode^3^ | 392 | 2.5 | 2.3-2.9 | 75 | 13.2 | 10.4-16.7 |
| Agoraphobia without panic (f40.00) | 84 | 0.6 | 0.4-0.7 | 21 | 3.6 | 2.3-5.7 |
| Agoraphobia with panic (f40.01) | 78 | 0.5 | 0.4-0.7 | 23 | 4.1 | 2.5-6.6 |
| Social phobia (f40.1) | 146 | 0.9 | 0.8-1.1 | 38 | 6.4 | 4.5-9.1 |
| Specific (isolated) phobia (f40.2) | 108 | 0.7 | 0.6-0.9 | 29 | 5.1 | 3.3-7.6 |
| Any phobia^4^ | 252 | 1.7 | 1.5-2.0 | 66 | 11.8 | 9.1-15.2 |
| Panic disorder (f41.0) | 100 | 0.8 | 0.6-1.0 | 11 | 2 | 1.0-3.7 |
| Generalised anxiety disorder (f41.1) | 646 | 4.5 | 4.1-4.9 | 93 | 16.6 | 13.2-20.7 |
| Obsessive compulsive disorder (f42) | 147 | 1.1 | 0.9-1.3 | 25 | 4.1 | 2.5-6.8 |
| CMD Not Otherwise Specified (f41.2) | 1036 | 7.6 | 7.1-8.1 | 106 | 17.3 | 14.2-20.8 |
| **Data are shown as raw sample n and survey weighted proportions. Pearson’s X^2^ p-value <0.0001 unless otherwise indicated.*  ^1^*p=0.005.*  *^2^International Classification for Diseases-10 diagnosis code, derived through the Clinical Interview Schedule-Revised.*  *^3^Combined mild depressive episode, moderate depressive episode, and severe depressive episode.*  *^4^Combined agoraphobia without panic, agoraphobia with panic, social phobia, specific (isolated) phobia.* | | | | | | |

# 3. Quasi-proportional Euler diagrams illustrating overlaps between health problems for the formerly homeless group and never homeless group*.

*All proportions are survey-weighted; proportion sizes in the diagram are approximate and were generated using the Eulerr web tool (Larsson and Gustafsson, 2018, Larsson, 2021).


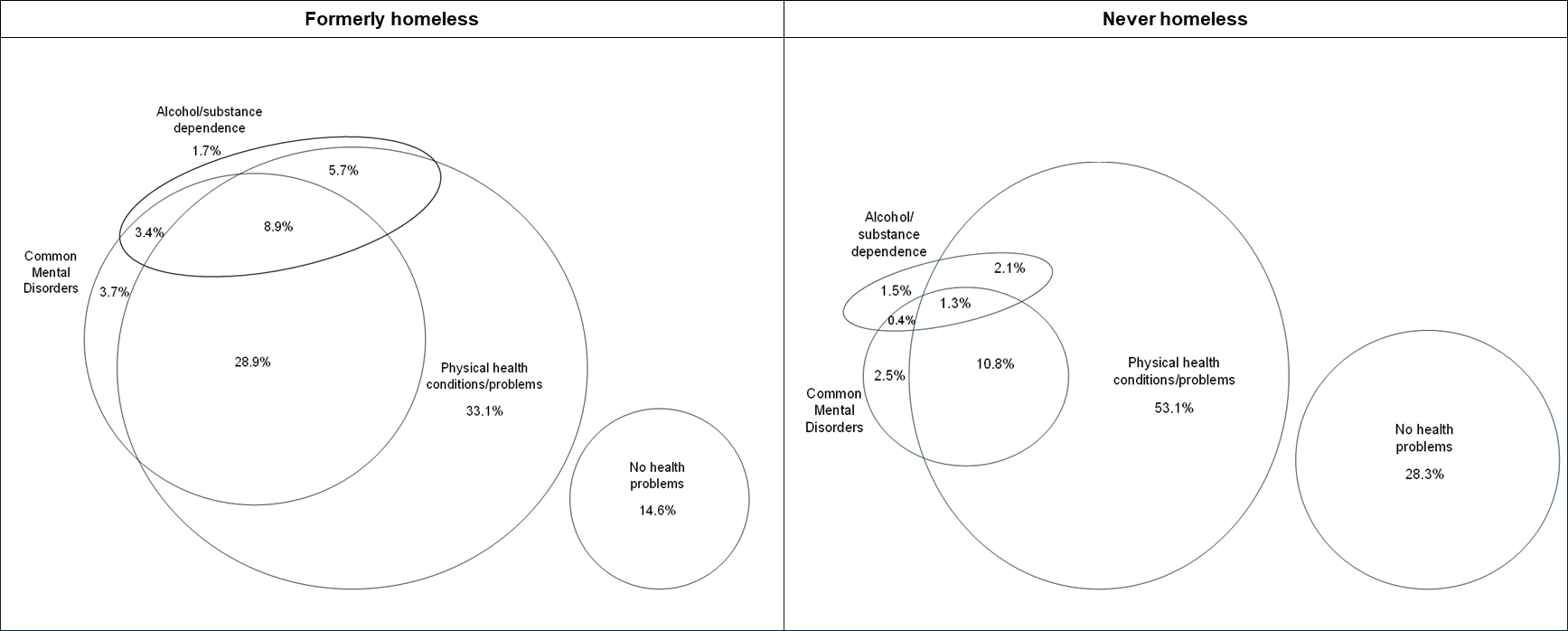


# 4. STROBE checklist for observational studies.

STROBE Statement—checklist of items that should be included in reports of observational studies.

|  | Item No. | Recommendation | Page  No. |
| --- | --- | --- | --- |
| **Title and abstract** | 1 | (*a*) Indicate the study’s design with a commonly used term in the title or the abstract | 3 |
|  |  | (*b*) Provide in the abstract an informative and balanced summary of what was done and what was found | 3 |
| Introduction | | | |
| Background/rationale | 2 | Explain the scientific background and rationale for the investigation being reported | 3-4 |
| Objectives | 3 | State specific objectives, including any prespecified hypotheses | 4 |
| Methods | | | |
| Study design | 4 | Present key elements of study design early in the paper | 4 |
| Setting | 5 | Describe the setting, locations, and relevant dates, including periods of recruitment, exposure, follow-up, and data collection | 4-5 |
| Participants | 6 | (*a*) *Cohort study*—Give the eligibility criteria, and the sources and methods of selection of participants. Describe methods of follow-up  *Case-control study*—Give the eligibility criteria, and the sources and methods of case ascertainment and control selection. Give the rationale for the choice of cases and controls  *Cross-sectional study*—Give the eligibility criteria, and the sources and methods of selection of participants | 4 |
|  |  | (*b*) *Cohort study*—For matched studies, give matching criteria and number of exposed and unexposed  *Case-control study*—For matched studies, give matching criteria and the number of controls per case | n/a |
| Variables | 7 | Clearly define all outcomes, exposures, predictors, potential confounders, and effect modifiers. Give diagnostic criteria, if applicable | 4-5 |
| Data sources/ measurement | 8* | For each variable of interest, give sources of data and details of methods of assessment (measurement). Describe comparability of assessment methods if there is more than one group | 4-5 |
| Bias | 9 | Describe any efforts to address potential sources of bias | 6 |
| Study size | 10 | Explain how the study size was arrived at | Figure 1 |

Continued on next page

| Quantitative variables | 11 | Explain how quantitative variables were handled in the analyses. If applicable, describe which groupings were chosen and why | 4-5 |
| --- | --- | --- | --- |
| Statistical methods | 12 | (*a*) Describe all statistical methods, including those used to control for confounding | 6 |
|  |  | (*b*) Describe any methods used to examine subgroups and interactions | 6 |
|  |  | (*c*) Explain how missing data were addressed | 6, Appendix 1, Figure 1 |
|  |  | (*d*) *Cohort study*—If applicable, explain how loss to follow-up was addressed  *Case-control study*—If applicable, explain how matching of cases and controls was addressed  *Cross-sectional study*—If applicable, describe analytical methods taking account of sampling strategy | 6 |
|  |  | (*e*) Describe any sensitivity analyses | n/a |
| Participants | 13* | (a) Report numbers of individuals at each stage of study—eg numbers potentially eligible, examined for eligibility, confirmed eligible, included in the study, completing follow-up, and analysed | Figure 1 |
|  |  | (b) Give reasons for non-participation at each stage | Figure 1 |
|  |  | (c) Consider use of a flow diagram | Figure 1 |
| Descriptive data | 14* | (a) Give characteristics of study participants (eg demographic, clinical, social) and information on exposures and potential confounders | 6-7, Table 1 |
|  |  | (b) Indicate number of participants with missing data for each variable of interest | Appendix 1 |
|  |  | (c) *Cohort study*—Summarise follow-up time (eg, average and total amount) | n/a |
| Outcome data | 15* | *Cohort study*—Report numbers of outcome events or summary measures over time | n/a |
|  |  | *Case-control study—*Report numbers in each exposure category, or summary measures of exposure | n/a |
|  |  | *Cross-sectional study—*Report numbers of outcome events or summary measures | 7-8, Table 2, Appendix 2, Appendix 3 |
| Main results | 16 | (*a*) Give unadjusted estimates and, if applicable, confounder-adjusted estimates and their precision (eg, 95% confidence interval). Make clear which confounders were adjusted for and why they were included | 7-8, Table 3, Table 4 |
|  |  | (*b*) Report category boundaries when continuous variables were categorized | Table 2, Table 3, Table 4 |
|  |  | (*c*) If relevant, consider translating estimates of relative risk into absolute risk for a meaningful time period | n/a |

Continued on next page

| Other analyses | 17 | Report other analyses done—eg analyses of subgroups and interactions, and sensitivity analyses | 6 |
| --- | --- | --- | --- |
| Key results | 18 | Summarise key results with reference to study objectives | 8 |
| Limitations | 19 | Discuss limitations of the study, taking into account sources of potential bias or imprecision. Discuss both direction and magnitude of any potential bias | 8-9 |
| Interpretation | 20 | Give a cautious overall interpretation of results considering objectives, limitations, multiplicity of analyses, results from similar studies, and other relevant evidence | 9 |
| Generalisability | 21 | Discuss the generalisability (external validity) of the study results | 9 |
| Other information | |  |  |
| Funding | 22 | Give the source of funding and the role of the funders for the present study and, if applicable, for the original study on which the present article is based | 10 |

*Give information separately for cases and controls in case-control studies and, if applicable, for exposed and unexposed groups in cohort and cross-sectional studies.

**Note:** An Explanation and Elaboration article discusses each checklist item and gives methodological background and published examples of transparent reporting. The STROBE checklist is best used in conjunction with this article (freely available on the Web sites of PLoS Medicine at http://www.plosmedicine.org/, Annals of Internal Medicine at http://www.annals.org/, and Epidemiology at http://www.epidem.com/). Information on the STROBE Initiative is available at [www.strobe-statement.org](https://url.avanan.click/v2/___http://www.strobe-statement.org___.YXAxZTpjYW1icmlkZ2Vvcmc6YTpvOmY3NGRmMzExNmJjNzRlZjQ5YTMxYjI4YmFmNmFmNjE0OjY6YTczNDo5MGZjYTY4NTljMGM1NjZlNDE1MzAzMTEyYzNlMTgzNjY0ZDI4ZjZkMmVjYTRjMDgxNmU5ODk1NmJlMmUzZDU3OnA6VDpG)

# 5. Appendix references

**Larsson, J.** 2021. *eulerr: Area-Proportional Euler and Venn Diagrams with Ellipses.* [Online]. R Package version 6.1.1. Available: [https://CRAN.R-project.org/package=eulerr](https://url.avanan.click/v2/___https://CRAN.R-project.org/package=eulerr___.YXAxZTpjYW1icmlkZ2Vvcmc6YTpvOmY3NGRmMzExNmJjNzRlZjQ5YTMxYjI4YmFmNmFmNjE0OjY6ZjkyMjozNTU5YzhjNDIzZjljMDRhN2RkOThlYWU3ZDlmMGEyYzI4YWM0MDBiNjI5MGJhOTBjODY1YzAwYjJmN2NlNzIzOnA6VDpG) [Accessed].

**Larsson, J. & Gustafsson, P.** A case study in fitting area-proportional euler diagrams with ellipses using eulerr. SetVR@ Diagrams, 2018. 84-91.
